# The acute effects of growth hormone in adipose tissue is associated with suppression of antilipolytic signals

**DOI:** 10.14814/phy2.14373

**Published:** 2020-02-19

**Authors:** Katrine L. Høyer, Morten L. Høgild, Edward O. List, Kevin Y. Lee, Emily Kissinger, Rita Sharma, Nils Erik Magnusson, Vishwajeet Puri, John J. Kopchick, Jens O. L. Jørgensen, Niels Jessen

**Affiliations:** ^1^ Medical Research Laboratory Department of Clinical Medicine, Health Aarhus University Aarhus Denmark; ^2^ Department of Endocrinology Aarhus University Hospital Aarhus Denmark; ^3^ The Edison Biotechnology Institute Athens OH USA; ^4^ Heritage College of Osteopathic Medicine Ohio University Athens OH USA; ^5^ Department of Clinical Pharmacology University of Aarhus Aarhus Denmark; ^6^ Department of Biomedicine Aarhus University Aarhus Denmark; ^7^ Steno Diabetes Center Aarhus Aarhus University Hospital Aarhus Denmark

**Keywords:** clinical studies, fatty acid/metabolism, gene expression, growth hormone, hormones, lipase/hormone sensitive

## Abstract

**Aim:**

Since GH stimulates lipolysis in vivo after a 2‐hr lag phase, we studied whether this involves GH signaling and gene expression in adipose tissue (AT).

**Methods:**

Human subjects (*n* = 9) each underwent intravenous exposure to GH versus saline with measurement of serum FFA, and GH signaling, gene array, and protein in AT biopsies after 30–120 min. Human data were corroborated in adipose‐specific GH receptor knockout (FaGHRKO) mice versus wild‐type mice. Expression of candidate genes identified in the array were investigated in 3T3‐L1 adipocytes.

**Results:**

GH increased serum FFA and AT phosphorylation of STAT5b in human subjects. This was replicated in wild‐type mice, but not in FaGHRKO mice. The array identified 53 GH‐regulated genes, and Ingenuity Pathway analysis showed downregulation of *PDE3b*, an insulin‐dependent antilipolytic signal, upregulation of *PTEN* that inhibits insulin‐dependent antilipolysis, and downregulation of *G0S2* and *RASD1,* both encoding antilipolytic proteins. This was confirmed in 3T3‐L1 adipocytes, except for *PDE3B*, including reciprocal effects of GH and insulin on mRNA expression of *PTEN, RASD1*, and *G0S2.*

**Conclusion:**

**(**a) GH directly stimulates AT lipolysis in a GHR‐dependent manner, (b) this involves suppression of antilipolytic signals at the level of gene expression, (c) the underlying GH signaling pathways remain to be defined.

## Graphical abstract

Our data demonstrate that a physiological GH pulse:1.Evokes acute in vivo GH signaling in human adipose tissue 2. Stimulates lipolysis after a lag phase of 2 hr and regulates expression of genes involved in antilipolysis 3. This was supported by mice and in vitro models, which suggest that GH acts as an insulin‐antagonistic hormone to favor lipolysis by releasing of the brakes.

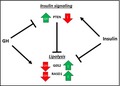

## 1 INTRODUCTION

Triglycerides (TAG) in adipose tissue, which constitute the major energy depot in the body (Large, Peroni, Letexier, Ray, & Beylot, 2004) are hydrolyzed to free fatty acids (FFA) and glycerol during catabolic conditions by a hormonally regulated process called lipolysis (Nielsen, Jessen, Jorgensen, Moller, & Lund, 2014; Wolfe et al., 1987). Catecholamines are the primary stimulators of lipolysis via activation of β‐adrenergic receptors on adipocytes leading to increased cAMP and activation of protein kinase A (PKA) (Lafontan & Berlan, 1993). PKA activates the hormone sensitive lipase (HSL) and perilipin, which facilitates access to substrates on the lipid droplets and initiate lipolysis (Nielsen, Jessen, Jorgensen, Moller, & Lund, 2014).

GH is also a recognized regulator of lipolysis (Moller & Jorgensen, 2009; Raben, 1962), and we have shown that an intravenous GH bolus induces a several fold increase in circulating FFA levels peaking after 2–3 hr ( Moller, Jorgensen, Schmitz, et al., 1990) followed by a gradual decline toward baseline after 8 hr (Gravholt et al., 1999). This is accompanied by and causally linked to suppression of insulin‐stimulated glucose uptake in skeletal muscle,(Moller, Jorgensen, Alberti, Flyvbjerg, & Schmitz, 1990), which is noteworthy since insulin is also a main inhibitor of lipolysis (Jensen & Nielsen, 2007).

The exact molecular mechanisms underlying the lipolytic effects of GH, however, remain uncertain. In a previous study, we investigated the acute effect of an intravenous GH pulse on gene expression in skeletal muscle in healthy subjects using a gene array approach (Clasen et al., 2013). The regulation of known GH‐dependent genes was detected concomitant with an acute increase in circulating FFA levels, but genes associated with lipid mobilization in skeletal muscle were not changed. This suggests that the lipolytic effect of GH is due to its direct effects on adipose tissue.

In the present study, we tested the hypothesis that a single GH pulse acutely regulates the expression of mRNA associated with lipolysis in adipose tissue (AT). To this end, eight subjects underwent AT biopsies before and two hours after administration of an intravenous GH bolus. This was complemented by mechanistic studies in mice and cells.

## 2 RESULTS

### 2.1 GH acutely stimulates pSTAT5 phosphorylation in adipose tissue and increases serum FFA levels in human subjects

**Figure 1:**
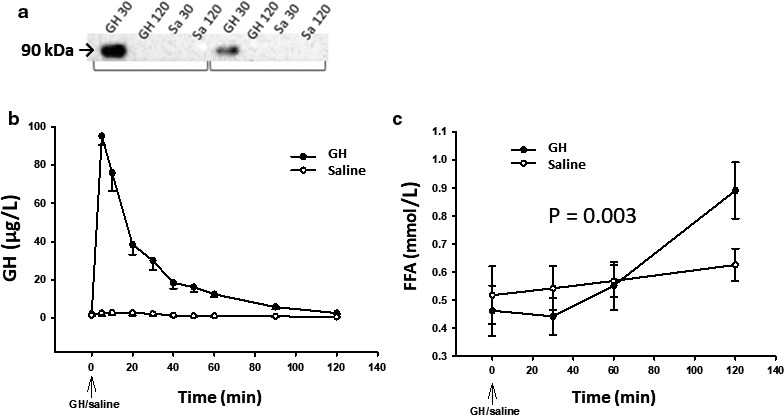
Panel (a) two representative Western blots of phosphorylated STAT5 30 and 120 minutes after intravenous bolus of GH and saline using anti‐STAT5 antibody from Cell Signaling Technology (Cat. No.: #4322). Lower panel: mean ± SE levels of serum GH (a) and serum FFA (b) after exposure to GH (filled circles) and saline (open circles). (*p* = .003)

Phosphorylation of STAT5 (pSTAT5) 30 min after GH was detected in seven of nine subjects (Figure 1a), but no pSTAT5 bands were detected 120 min after either GH or saline. Data on circulating GH and FFA levels from the entire study population have been reported previously (Vestergaard et al., 2014); in this study, we extracted data from the eight subjects, who contributed to microarray analysis. GH peaked in serum 5 min after its administration [95.1 ± 4.9 ug/l (mean ± SE)], and returned to baseline after 120 min (Figure 1b). Serum FFA levels increased 120 min after the GH bolus by >40 percentage compared to the saline study (*p* = .003) (Figure 1c).

### 2.2 GH acutely increases serum FFA levels in a GHR‐dependent manner in adipose tissue in mice

**Figure 2:**
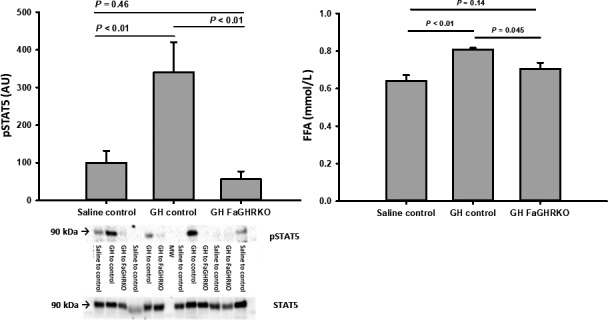
Phosphorylation of STAT5 relative to total STAT5 in subcutaneous adipose tissue (a) and serum FFA levels (b) in wild‐type mice (control) and GH receptor knock‐out mice (FaGHRKO) 120 min after intraperitoneal administration of saline or GH. Representative western blots are shown under using anti‐STAT5 antibody from Cell Signaling Technology (Cat. No.: #4322) and anti‐STAT5 antibody from AbCam (# ab194898 ) (a). STAT5 phosphorylation was only detected after GH administration in control mice

To substantiate that the elevation in circulating FFA after acute GH exposure originates from adipose tissue, bGH was injected both in wild‐type mice and in FaGHRKO followed by measurements of pSTAT5b in adipose tissue and FFA levels in serum. GH‐induced STAT5 phosphorylation as well as elevation of serum FFA levels occurred in wild‐type mice only (Figure 2). The GH‐induced GH response did not differ between male and female mice (*p* = .59).

### 2.3 GH acutely regulates mRNA expression in human adipose tissue

**Figure 3:**
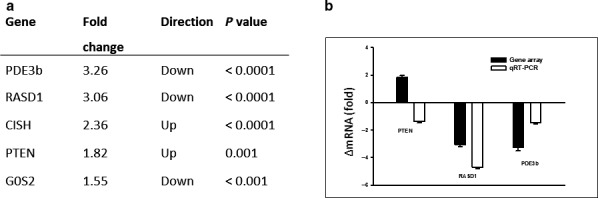
(a): Microarray data for genes involved in GH signaling and lipolysis. Differentially expressed genes 120 minutes after stimulation with GH compared to the GH unstimulated state. Note: the presented p values are unadjusted; for further information see results (b): Lower panel: Comparative mRNA determinations of selected target genes with the microarray (open bars) and qRT‐PCR (filled bars). Data are presented as mean ± SE of fold change in mRNA expression of GH exposed samples relative to GH unexposed samples

The microarray analysis identified 53 transcript clusters that were differentially expressed in adipose tissue 120 min after GH compared to saline (Appendix). Supervised hierarchical cluster analysis based on unadjusted significant results separated two distinct clusters based on GH intervention (data not shown). The expression of each of the 53 transcript clusters changed at least 1.5 fold (*p* < .05 (unadjusted)) compared to the unstimulated state. The regulated genes were analyzed using Ingenuity Pathway analysis (Qiagen). Genes related to metabolic functions and the lipolytic signaling pathway were a priori considered of special interest (Figure 3a). When using the Benjamini–Hochberg correction for adjusted p value, only the transcription of *PDE3B* (*p* = .05) and *RASD1* (*p* = .01) remained significantly different after GH exposure. To validate the data from the microarray, mRNA levels of 3 genes (*PTEN*, *RASD1* and *PDE3B*) were measured by Real‐time PCR, which yielded concurrent results with regard to RASD1 and PDE3B, but not with regard to *PTEN* (Figure 3b).

### 2.4 GH directly regulates the mRNA expression of GH‐dependent genes and genes involved in regulation of lipolysis in 3T3‐L1 adipocytes in vitro

**Figure 4:**
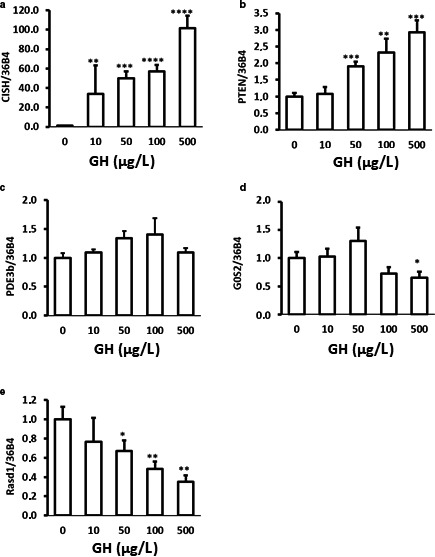
qPCR analysis of PTEN, RASD1, CISH, PDE3B, and G0S2 mRNA abundance isolated from 3T3‐L1 adipocytes treated with bGH (GH) for 2 hr. Data are shown as mean ± SE of three independent experiments

To test if GH could directly regulate the mRNA levels of the lipolytic target genes identified in vivo, we incubated 3T3‐L1 adipocytes with GH. As depicted in Figure 4, GH dose‐dependently increased the expression of *CISH*, a bona‐fide STAT5 regulated gene, together with a dose‐dependent increase in the transcription of *PTEN,* whereas *RASD1* and *G0S2* were negatively regulated. We did not observe consistent GH‐dependent regulation of *PDE3B* under these conditions (Figure 4c).

### 2.5 Insulin and GH regulates mRNA expression in a reciprocal manner

**Figure 5:**
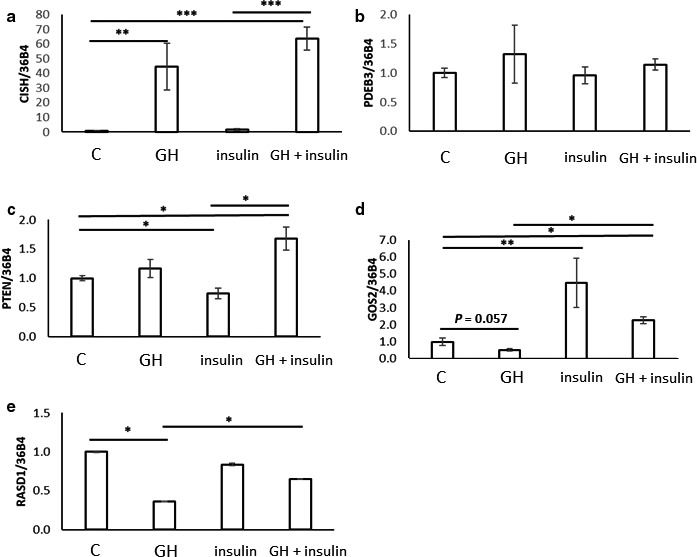
qPCR analysis of CISH, PDE3B, PTEN, G0S2, and RASD1 mRNA abundance isolated from 3T3‐L1 adipocytes with no treatment (C), treated with 500 µg/l bGH (GH) and/or 100 nmol/l insulin for 2 hr. Data are shown as mean ± SE of three independent experiments. *p; .05, **p; .01, ***p; .001

Since GH and insulin exhibit antagonistic actions in the regulation of lipolysis, we incubated 3T3‐L1 adipocytes with insulin and GH alone and in combination. Insulin alone had no effect on the expression of *CISH* or *PDE3B* (Figure 5a,b)*.* By contrast, *PTEN* mRNA levels were repressed ≈30% by insulin and this was abrogated by GH (Figure 5c). Furthermore, insulin treatment lead to an ≈ fivefold increase in the expression of *G0S2* mRNA, whereas GH alone tended to do the opposite (Figure 5d). Co‐administration of GH and insulin reduced G0S2 mRNA expression as compared to insulin alone, albeit not significantly (Figure 5d). GH suppressed RASD1 mRNA, which was antagonized by insulin (Figure 5e).

## 3 DISCUSSION

The present study demonstrates that acute stimulation of lipolysis and GHR signaling in human AT are detectable in vivo after an intravenous bolus of GH. This was documented by a significant increase in circulating FFA levels concomitantly with AT STAT5b phosphorylation, which is the upstream part of the major GH signaling pathway. This observation was validated in mice experiments by the absence of STAT5b phosphorylation and FFA release in FaGHRKO mice during GH stimulation. At the level of gene expression, our main finding was that GH acutely activates several genes promoting inhibition of antilipolytic signals, which we confirmed in vitro in adipocytes incubated with GH.

The mRNA expression of cytokine‐inducible SH2 protein (CISH) was upregulated in the microarray and highly indicative of GH‐induced STAT5b signaling as previously documented (Clasen et al., 2014).

The observed downregulation of PDE3b mRNA expression after GH exposure is interesting, since PDE3b is an insulin‐dependent negative regulator of lipolysis via degradation of cAMP (Degerman et al., 1998; DiPilato et al., 2015). We observed no GH‐induced change in PDE3b mRNA expression in our in vitro model with 3T3‐L1 adipocytes, so it remains a possibility that the human in vivo data derive from nonadipocyte cells such as endothelial cells or fibroblasts. With the array, we also observed GH‐induced upregulation of the PTEN gene, although this was not replicated with PCR; PTEN is a phosphatase that inhibits insulin signaling via dephosphorylation of PIP_3_ (Taniguchi, Emanuelli, & Kahn, 2006). Taken together, the coordinated regulation of these genes may contribute to GH‐induced insulin resistance.

The RASD1 gene, which was downregulated by GH, encodes the Dexras1 protein, a member of the steroid hormone‐induced Ras family G‐protein that ultimately inhibits lipolysis via a cAMP dependent mechanism (Graham, Qiao, & Dorin, 2004; Kemppainen & Behrend, 1998). We also found downregulation of G0S2 that is a lipid droplet (LD)‐associated inhibitor of adipose triglyceride lipase (ATGL) in adipocytes (Nielsen & Moller, 2014). Previous studies have shown decreased G0S2 during fasting (Nielsen et al., 2011) and positively regulated by insulin (Yang et al., 2010), which is noteworthy since GH‐induced lipolysis mainly operate during fasting.

**Figure 6:**
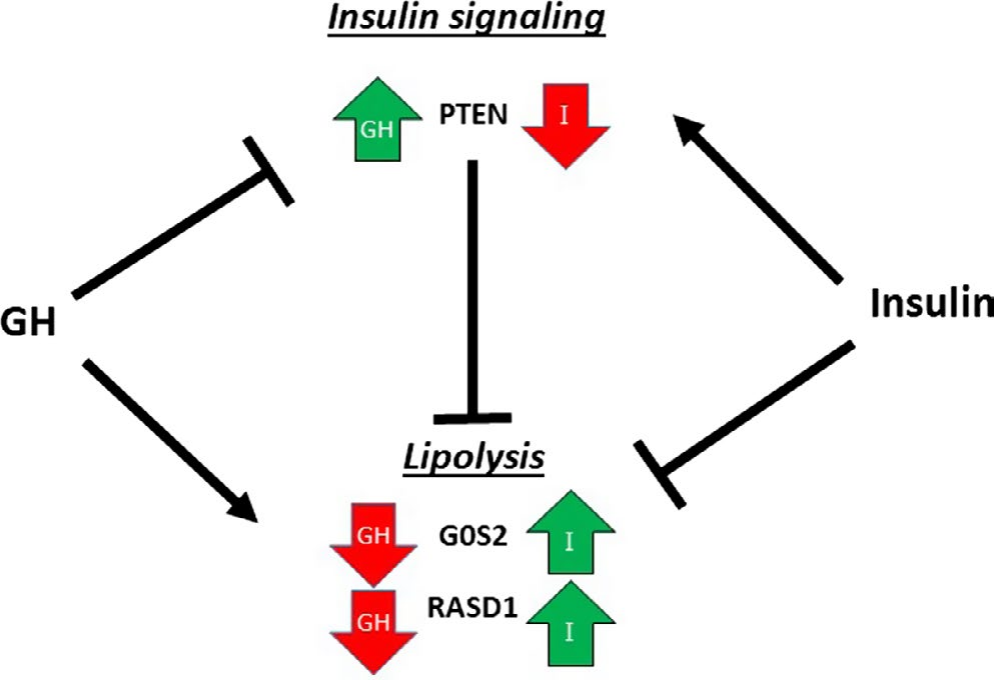
Schematic model and interpretation of the data regarding the interaction between GH and insulin actions on lipolytic regulators in adipose tissue. GH upregulates PTEN mRNA, which suppresses insulin signaling and thereby insulin‐mediated anti‐lipolysis. GH downregulates mRNA expression of G0S2 and RASD1 both of which suppress lipolysis in an insulin‐dependent manner. Put together, GH predominantly suppresses anti‐lipolytic signal

In further support of the notion that GH‐induced lipolysis is characterized by suppression of antilipolysis, we recorded reciprocal effects of GH and insulin on mRNA expression of *G0S2*, *RASD1*, and *PTEN*. Moreover, GH abrogated the effects of insulin on PTEN mRNA expression, whereas insulin antagonized the effects of GH on G0S2 and RASD1 mRNA. Taken together, these data suggest that GH‐induced lipolysis depends on suppression of antilipolytic signals including insulin, and as such that GH‐induced insulin resistance may be necessary for its lipolytic effects (Figure 6).

We recently reported that GH downregulates the mRNA and protein expression of fat specific protein 27 (FSP27), also known as CIDEC, in studies involving GH infusion with and without fasting in healthy young male subjects (mean age ≈ 21 years) (Sharma et al., 2018), as well as in studies in mice(Sharma et al., 2018) and in human adipocytes in vitro (Sharma et al., 2019). Fat‐specific protein 27 is an LD‐associated protein that regulates LD dynamics (Jambunathan, Yin, Khan, Tamori, & Puri, 2011; Puri et al., 2007) and lipolysis in adipocytes through downregulation of the transcription and activity of ATGL, the rate‐limiting enzyme in lipolysis (Singh et al., 2014). In the present human study, FSP27 was insignificantly (*p* = .18) downregulated by 1.09 fold after GH (data not shown) without any apparent gender effect; whether the discrepant results relate to mode of GH exposure or age of the subjects remains to be tested.

It also remains to be investigated how the GH‐dependent genes in the present study are being transcribed, in particular whether it depends on STAT5 or alternative pathways of GH signaling.

Our data contrasts with a gene array study performed in GH‐deficient male patients studied before and after one month of GH replacement that recorded mRNA downregulation of *CIDEA*, a lipid droplet protein, and upregulation of *PNPLA3,* a novel TG hydrolase (Zhao et al., 2011), both of which were not significantly regulated by GH in our study. The discrepancy may relate to the difference in design between the two studies.

Some methodological aspects merit attention. First, we used the rise in serum FFA levels as a biomarker of GH‐induced lipolysis in adipose tissue in vivo, which may lack both sensitivity and specificity. However, we and others have consistently documented the lipolytic effects of GH in vivo by means of more precise measures including glycerol concentrations in serum (Moller, Jorgensen, Alberti, et al., 1990) as well as in the interstitial fluid by means of microdialysis (Gravholt et al., 1999 **),** and we have also shown that GH increases fatty acid turnover assessed by tracer techniques (Kanaley et al., 2004; Krag et al., 2007; Norrelund et al., 2003). In recent cell studies, we have also demonstrated that GH acutely stimulates glycerol release (Sharma et al., 2018, 2019). Second, it is inherently difficult to combine human in vivo studies with animal and in vitro models. Our pivotal experiment aimed to study the acute effect of a GH bolus on mRNA expression in human adipose tissue in vivo. To gain further mechanistic insight, we then performed studies in mice, but it is likely that species‐specific differences exist with regard to the physiological role and lipolytic effect of GH (Steyn et al., 2012). Third, supra physiological GH doses are required in most rodent and in vitro experiments including ours in order to elicit a response. This may weaken the external validity, but in general, we found good agreement between the human data and those obtained in vitro.

In summary, this study enabled detection of acute GH signaling in adipose tissue in vivo*,* which significantly regulated the expression of several genes involved in lipolysis and antilipolysis. Subsequent experiments in mice models and cultured adipocytes support that these effects are due to direct effects of GH on the adipocyte. We suggest that GH primarily acts by suppressing antilipolytic signals at the level of gene transcription. Future studies are needed to elucidate the underlying mechanisms in more detail.

## 4 MATERIALS AND METHODS

The human data derive from a clinical experiment from which data of a different nature have been published (Vestergaard et al., 2014). All subjects were studied in random order on two occasions after an overnight fast for 12 hr receiving an intravenous bolus of either GH (0.5 mg (Genotropin miniquick, Pfizer)) or saline. Subcutaneous adipose tissue biopsies from the periumbilical region were obtained 30 and 120 min after administration of GH/saline and accompanied by frequent blood sampling. The biopsies obtained 120 min after GH/saline were used for microarray and qRT‐PCR analysis while both biopsies were studied for protein analysis. The original study included 20 subjects but due to limited availability of AT samples, gene expression was measured in 8 subjects [two males and six females with a mean (range) age: 35 (24; 64) years, and mean ± SE body mass index: 25.4 ± 1.3 kg/m^2^], whereas protein expression was measured in a different subset consisting of three males and six females. The protocol was approved by the Ethical Committee of Central Denmark Region, and the study was conducted in agreement with the Helsinki Declaration.

### 4.1 Serum sampling and biopsies from the human subjects

Serum samples from the human experiments were collected consecutively during each study day and stored at −20°C. Insulin and GH levels were measured using time‐resolved fluoroimmunoassay (TR‐IFMA, AutoDELFIA; PerkinElmer) and FFA levels were analyzed with a commercial kit (Wako Chemicals). Under sterile conditions and local anesthesia (Lidokain: Amgros), AT biopsies were obtained from periumbilical subcutaneous fat by liposuction with a 50 ml syringe. The biopsies were subsequently cleaned for visible blood with RNA free saline, snap frozen in liquid nitrogen and stored at −80˚C until analyses were performed.

### 4.2 GH‐stimulation of adipose‐specific GH receptor knockout mice

FaGHRKO were generated by breeding conditional GHR flox/flox mice to B6.Cg‐Tg(Fabp4‐cre)1Rev/J mice as previously described (List et al., 2013). Detailed analysis of the Fabp4‐Cre mouse model indicates that it recombines alleles effectively in the brown and white fat pads and although there is some “off‐target” recombination in endothelial cells of the heart and nonendothelial, nonmyocyte cells in the skeletal muscle, it does effectively ablate gene expression in a relatively adipose‐specific manner( Lee et al., 2013). In the current study, a total of twelve mice (3M/8F) at eight months of age were divided into 3 groups: (a) five (4F/1M) wild‐type mice injected with saline (Control), (b) three wild‐type mice injected with GH (2F/1M), and (c) four (3F/1M) FaGHRKO mice injected with GH. The average weight of the mice was 32.3 ± 2.4 grams for wild‐type mice and 36.4 ± 3.1 grams for FaGHRKO mice. Mice in groups 2 and 3 were injected intraperitoneally (IP) with bGH (rBGH, CYT‐636, Prospec) at a dose of 6 μg/g body weight while the control mice received a vehicle (equal volume of isotonic 0.9% NaCl). Mice were fasted for 240 min prior to treatment. 120 min after injection with bGH/saline the mice were anesthetized, exsanguinated, and then sacrificed. Fasting and injections were staggered every 7 min to allow tissue collection to occur precisely 240 and 120 min after initiation of fasting and injections, respectively. Inguinal subcutaneous AT was removed and snap‐frozen in liquid nitrogen and serum samples were collected and stored. Mice were housed in groups of three to four per cage and given ad libitum access to water and standard laboratory rodent chow (ProLab RMH 3,000). The cages were maintained in a temperature‐ and humidity‐controlled room and exposed to a 14‐hr light, 10‐hr dark cycle. All procedures were approved by the Ohio University Institutional Animal Care and Use Committee.

### 4.3 RNA isolation and quantitative PCR in the human study

RNA was extracted from the AT biopsies using Trizol reagent (Life technologies Inc.). The amount and purity of total RNA was quantified by measuring optical density at 260 and 280 nm using a NanoDrop 8,000 spectrophotometer (Thermo Fisher Scientific, Inc., Waltham, MA, USA). Integrity of the RNA was checked by visual inspection of ribosomal RNAs, 18S and 28S, on an agarose gel. cDNA was synthesized using the Verso cDNA Kit AB 1,453 (Thermo Fisher Scientific, Inc.) using random hexamer primers. β2‐microglobulin expression levels were stable across experiments and this gene was used as the internal control for quantitative PCR. The following primers were used: β2‐microglobulin: 5′‐AATGTCGGATGGATGAAACC‐3′ and 5′‐TCTCTCTTTCTGGCCTGGAG‐3′; PTEN: 5′‐GACAGCCATCATCAAAGAGATCG‐3′ and 5′‐TCTGCAGGAAATCCCATAGCAA‐3′; PDE3b: 5′‐CACAATGGTTGTGGAACAGGAA‐3′ and 5′‐GACAGGCAGCCATAACTCTCA‐3′; RASD1: 5′‐CACAATGGTTGTGGAACAGGAA‐3′ and 5′‐GACAGGCAGCCATAACTCTCA‐3′; GAPDH: 5′‐GTTCGACAGTCAGCCGCAT and 5′‐TGACCAGGCGCCCAATAC‐3′; YWHAQ: 5′‐GGTACCTTGCTGAAGTTGCG‐3′ and 5′‐GGGTGTGTGGGTTGCATCT‐3′; GPX4: 5′‐CCGCTGTGGAAGTGGATGAA‐3′ and 5′‐ CACGCAGCCGTTCTTGTC‐3`.

### 4.4 Microarray analysis

RNA integrity number (RIN) score > 6 was obtained for all RNA samples. Total RNA (100 ng) was labeled with Ambion WT Expression Kit (Ambion) according to the manufacturer's instruction. Samples were hybridized overnight to the GeneChip Human Gene 2.0 ST Array (Affymetrix) and scanned using Affymetrix GCS 3,000 7G scanner. Data analysis were performed in the GeneSpring GX11.5 software (Agilent). Cell files were imported and quantile normalized with the iterPLIER16 algorithm followed by baseline transformation with median scaling to the median of all arrays. Data were deposited in NCBI’s Gene Expression Omnibus and are accessible through GEO accession number http://www.ncbi.nlm.nih.gov/geo/query/acc.cgi?acc=GSE124935.

### 4.5 Protein lysate preparation and Western blot analysis

Frozen AT biopsies were homogenized in solubilization buffer containing 50 mM HEPES, 20 mM NaF, 2 mM NaOV, 5 mM ESTA, 5 mM Nam, 10 µM TSA, protease inhibitor cocktail (HALT, Thermo Specific) and 5% SDS at pH 7,4. The samples were homogenized using a Precellys 24 homogenizer (Bertin Technologies) and kept on ice for 30 min and occasionally vortexed before centrifugation at 13,000 g for 20 min at 4°C for removal of insoluble material. The samples were denatured by mixing with 4x Lammili's buffer and heated at 95°C for 5 min. Western blot analysis were performed by SDS‐PAGE on StainFree 4%–15% TGX‐gels using the CriterionXT‐system from Bio‐Rad. Proteins were electro blotted onto PVDF membranes and the StainFree technique was used to control for loading( Gurtler et al., 2013). The membranes were then blocked for two hours in a 1% bovine serum albumin solution (Sigma‐Aldrich) and incubated with primary antibodies overnight. Antibodies directed against phosphorylated STAT5 were purchased from Cell Signaling Technology (Cat. No.: #4322), and STAT5 from AbCam (Cat. No.: # ab194898) were used. The following morning, the membranes were washed and incubated with secondary antibodies for two hours. Proteins on the membranes were visualized by ClarityTM ECL Western Blot analysis substrate (BioRad) and quantified with ChemiDocTM MP imaging system (BioRad). Protein Plus Precision All Blue standards were used as markers of molecular weight.

### 4.6 In vitro incubation of 3T3‐L1 adipocytes with GH and insulin

3T3‐L1 preadipocytes (ATCC) were maintained and differentiated into adipocytes as previously described (Lee, Gesta, Boucher, Wang, & Kahn, 2011). Differentiated adipocytes were treated with DMEM culture media containing 0, 10, 50, 100, or 500 ng/ml GH and/or 100 nM insulin (Sigma‐Aldrich) for two hours after which total RNA was isolated from the cells. Total RNA was isolated using TRIzol Reagent (Thermo Fisher). One μg of total RNA was reverse transcribed in 100 μl using the High Capacity cDNA Reverse Transcription Kit (Applied Biosystems). A portion (5 μl) of diluted (1/5) reverse transcription reaction was amplified with specific primers (300 nM each) in a 10 μl PCR using a SYBR green PCR master mix (Biorad). Analysis of individual gene expression was carried out in a Biorad CFX96 Touch™ Real‐Time PCR Detection System with initial denaturation at 95°C for 10 min, followed by 40 PCR cycles, each cycle consisting of 95°C for 15 s, 60°C for 1 min, and 72°C for 1 min, and SYBR green fluorescence emissions were monitored after each cycle. For each gene, mRNA expression was calculated relative to the 36B4 expression. Amplification of specific transcripts was confirmed by the melting‐curve profiles (cooling the sample to 68°C and heating slowly to 95°C with measurement of fluorescence) at the end of each PCR. The primers used for quantitative PCR are: mG0S2 5′‐ACTGACAGAGAAGGGAGACA‐3′ and 5′‐TTCGGTGGCACCTTGAAA‐3′; mPDE3b 5′‐GGTTCCTGTAGGCCAAAGATTA‐3′ and 5′‐GGAGTTGGGAAACTGGTTCT‐3′; mRasd1 5′‐GAAGGCTCTGAGGAACAAGAA‐3′ and 5′‐ATGTACATGAGGTCGCTGTG‐3′; mPTEN 5′‐ATTGCCTGTGTGTGGTGATA‐3′and 5′‐TCCTCTGGTCCTGGTATGAA‐3′; mCISH 5′‐CTCTGGGACATGGTCCTTTG‐3′ and 5′‐GTCACTTCCTCTGGGAATGC‐3′; and m36B4 5′GCAGACAACGTGGGCTCCAAGCAGAT‐3′ and 5′‐GGTCCTCCTTGGTGAACACGAAGCCC‐3′.

### 4.7 Statistical analysis

Sigmaplot 11.0 (Systat Software inc. for Windows) and Stata 13 (College Station) were used for statistical analysis. Comparisons among groups were done by Two‐Way‐ANOVA for repeated measures. When significant main effects or interactions were found, the Student–Newman–Keul's test was used for post hoc testing. A *p* < .05 was considered significant. The data on changes in gene expression from the microarray was log_2_ transformed. Fold change and the Student's paired *t*‐test (p value) were calculated. The data on protein expression in AT were not normally distributed and therefore tested using a Signed Rank paired test.
