# Supplementary material for: The acute effects of growth hormone in adipose tissue is associated with suppression of antilipolytic signals
Source: Physiol Rep. 2020 Feb 19;8(3):e14373. doi: 10.14814/phy2.14373 (PMC7029434; doi:10.14814/phy2.14373)
Supplement: Supplementary file 1 [file PHY2-8-e14373-s001.docx]

**Supplemental table 1. Transcript clusters regulated after GH stimulation**

| Gene Symbol | Direction | *P* (unadjusted) | Fold Change |
| --- | --- | --- | --- |
| OTTHUMG00000165339\|CTD-2530H12.2 | Down | < 0.001 | 3.63 |
| PDE3B | Down | < 0.001 | 3.26 |
| RASD1 | Down | < 0.001 | 3.06 |
| MIR604 | Up | < 0.001 | 2.57 |
| MIR938 | Up | 0.001 | 2.45 |
| CISH | Up | < 0.001 | 2.36 |
| THBS1 | Up | < 0.001 | 2.30 |
| ENPP2 | Up | < 0.001 | 2.20 |
| SLC1A3 | Up | < 0.001 | 2.05 |
| OTTHUMG00000018686\|RP11-380G5.2 | Up | 0.013 | 2.04 |
| ARID5B | Up | < 0.001 | 1.95 |
| C5orf30 | Up | < 0.001 | 1.94 |
| CEBPB | Down | < 0.001 | 1.84 |
| HYMAI | Up | < 0.001 | 1.84 |
| PTEN | Up | < 0.001 | 1.82 |
| RWDD2A | Up | 0.002 | 1.81 |
| GJA1 | Up | 0.007 | 1.80 |
| AKR1CL1 | Up | < 0.001 | 1.78 |
| C2CD2 | Down | < 0.001 | 1.76 |
| ADAMTS9 | Up | < 0.001 | 1.74 |
| YME1L1\|TRDJ2 | Up | 0.004 | 1.75 |
| SYBU | Up | < 0.001 | 1.74 |
| DUSP10 | Down | < 0.001 | 1.69 |
| FLRT2LOC100506718 | Up | < 0.001 | 1.67 |
| TWIST2 | Down | < 0.001 | 1.67 |
| AOC4 | Down | 0.004 | 1.66 |
| STL | Up | < 0.001 | 1.66 |
| TRDJ4 | Up | 0.012 | 1.65 |
| F3 | Up | 0.004 | 1.65 |
| HILPDA | Down | < 0.001 | 1.65 |
| LRRN3 | Up | 0.002 | 1.62 |
| PFKFB3 | Down | < 0.001 | 1.62 |
| MAP2K6 | Up | 0.004 | 1.61 |
| TMEM2 | Up | < 0.001 | 1.61 |
| C10orf10 | Up | 0.008 | 1.58 |
| GLUL | Up | < 0.001 | 1.58 |
| RNF217 | Up | 0.001 | 1.58 |
| ZFAND5 | Up | < 0.001 | 1.56 |
| G0S2 | Down | < 0.001 | 1.55 |
| NPY1R | Up | < 0.001 | 1.55 |
| OTTHUMG00000154827\|AC093382.1 | Down | 0.003 | 1.55 |
| CEBPA | Down | < 0.001 | 1.54 |
| FOSL2 | Up | < 0.001 | 1.54 |
| MIR4451 | Up | 0.049 | 1.54 |
| RNA5-8SP3 | Up | 0.007 | 1.54 |
| STARD13-IT1 | Up | 0.003 | 1.54 |
| RND3 | Up | < 0.001 | 1.53 |
| CTNNAL1 | Up | 0.003 | 1.52 |
| SLC39A14 | Up | < 0.001 | 1.52 |
| COX14 | Down | 0.003 | 1.51 |
| CSRP2 | Down | 0.002 | 1.51 |
| LOC100132813 | Up | 0.018 | 1.51 |
| BTG2 | Down | 0.003 | 1.50 |
